# Intramural duodenal hematoma and hemoperitoneum after endoscopic treatment in a patient with chronic renal failure on hemodialysis: a case report

**DOI:** 10.1186/1757-1626-2-9083

**Published:** 2009-11-24

**Authors:** Sungjin Chung, Cheol Whee Park, Hyun Wha Chung, Seok Joon Shin, Yoon Sik Chang

**Affiliations:** 1 Division of Nephrology, St. Mary's Hospital, The Catholic University of Korea, 62 Yeouido-dong, Yeongdeungpo-gu, Seoul 150-713, Republic of Korea; 2 Division of Nephrology, Seoul St. Mary's Hospital, The Catholic University of Korea, 505 Banpo-dong, Seocho-gu, Seoul 137-040, Republic of Korea; 3 Division of Nephrology, Incheon St. Mary's Hospital, The Catholic University of Korea, 665-8 Bupyeong 6-dong, Bupyeong-gu, Incheon 403-720, Republic of Korea

## Abstract

**Introduction:**

An intramural duodenal hematoma is a rare but recognized complication that usually develops after abdominal trauma, predominantly in children and young adults.

**Case Presentation:**

We report the case of a patient that developed an intramural duodenal hematoma after endoscopic treatment for a bleeding ulcer who also was undergoing conventional hemodialysis for chronic renal failure associated with lupus nephritis. A hemoperitoneum and near-total duodenal obstruction developed but spontaneously resolved with conservative treatment.

**Conclusion:**

Clinicians should be aware of such possible complications after endoscopic hemostasis in patients with coagulation disorders.

## Introduction

Endoscopic hemostatic procedures such as local injection of epinephrine, polidocanal and fibrin tissue adhesive onto the mucosa, and hemoclipping are commonly used for the treatment of bleeding ulcers, instead of the open surgical approaches used in the past [1]. Although the risks are usually considered minimal, there are reports that intramural duodenal hematomas can develop as a complication after diagnostic or therapeutic endoscopy, especially in patients susceptible to hemorrhage such as those with end-stage renal disease (ESRD) [1,2]. We report here the case of a patient that developed an intramural hematoma of the duodenum after endoscopic treatment for a bleeding ulcer using epinephrine injection and hemoclipping; the patient had end-stage renal failure due to lupus nephritis and was undergoing hemodialysis. Clinicians should be aware of such complications after endoscopic hemostasis in patients with coagulation disorders.

## Case presentation

A 30-year-old woman complained, during a hemodialysis session, of diarrhea and subsequent melena for one-day. The patient was on a schedule of hemodialysis three times a week at the dialysis unit of our hospital; she had chronic renal failure due to lupus nephritis, and was being treated with oral prednisolone, 20 mg daily, for control of lupus disease activity. In addition, the patient was usually treated with nafamostat mesilate as a hemodialysis anticoagulant.

**Figure 1:**
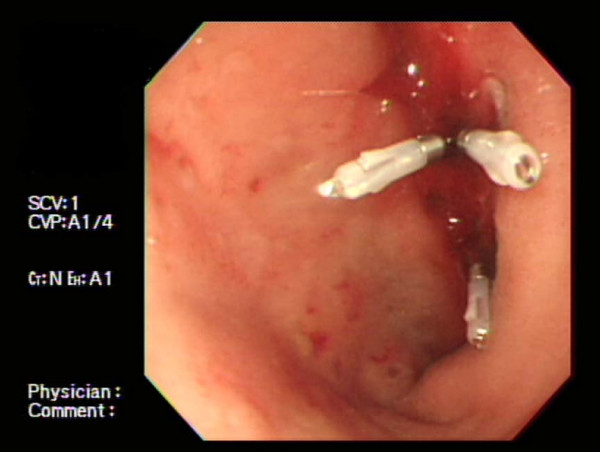
**Initial endoscopic findings revealed bleeding ulcers of the duodenum that were hemoclipped**.

At that time, a physical examination revealed anemic conjunctivae but no icteric sclera, abdominal tenderness or organomegaly. The blood pressure was 150/95 mmHg, pulse rate 88/min, and body temperature 36.4°C. Laboratory investigations on admission were as follows: hemoglobin 8.4 g/dL; white blood cell count 1320/mm^3^; platelet count 77,000/mm^3^; prothrombin 96.2% with INR 1.02; partial thromboplastin time 68.4 seconds; serum albumin 3.58 g/dL; serum aspartate transaminase (AST) 15 IU/L; serum alanine transaminase (ALT) 9 IU/L; alkaline phosphatase 123 IU/L; and total cholesterol 152 mg/dL. Hepatitis B surface antigen, hepatitis C antibody, and HIV antibody were all negative. Since the patient had been treated, at various times, with corticosteroids and pulse cyclophosphamide, the C3, C4, ANA and anti-ds DNA were normal; however, she had a positive antiplatelet antibody test. Because of the suspicion of active gastrointestinal bleeding, emergency gastrofiberscopy was performed immediately after the hemodialysis session. Endoscopy revealed two oozing ulcers in the duodenal wall at the duodenal bulb with exposed vessels and one round ulcer at the high body of the lesser curvature of the stomach. An injection of 0.2% epinephrine to a total of 2.5 mL with subsequent hemoclipping was performed for the duodenal ulcers with active bleeding (Figure 1). The patient was treated with intravenous administration of omeprazole. After the endoscopic hemostasis, the patient developed sharp epigastric pain, nausea and intermittent vomiting. The physicians considered the symptoms were due to the endoscopic procedure. The next day, the patient appeared pale, with a decreased hemoglobin level to 5.8 g/dL. Follow-up endoscopy showed no further bleeding evidence of the corresponding duodenal ulcers. Three days after the endoscopy, progression of the anemia was noted without hemodynamic instability; the patient required frequent transfusions.

**Figure 2:**
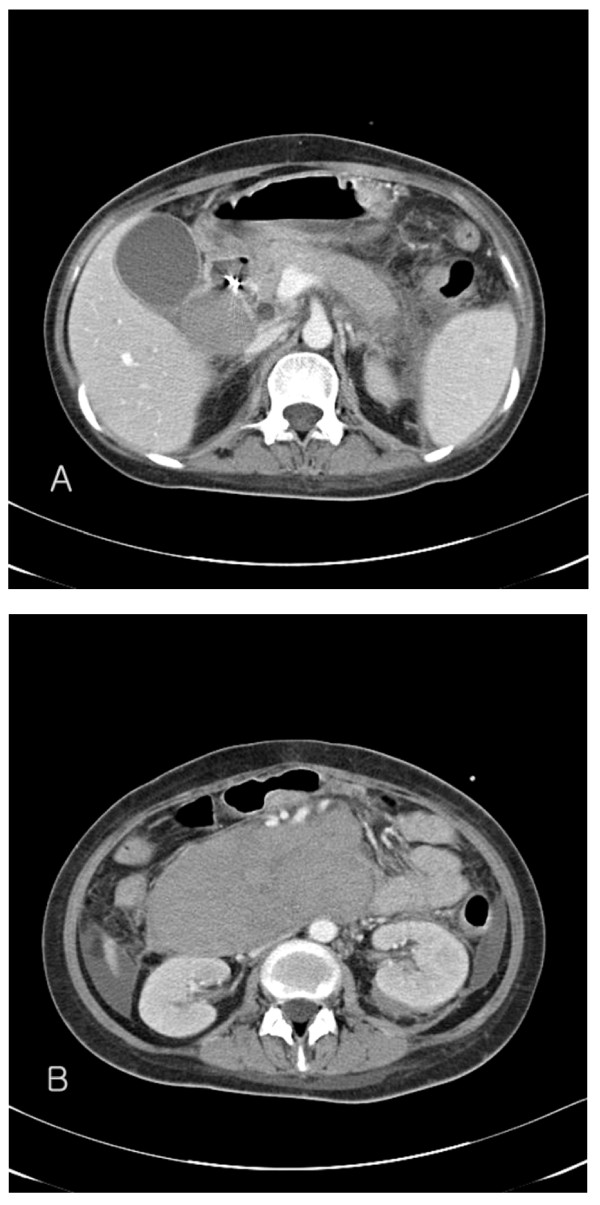
**CT of the abdomen demonstrates a mass with a filling defect in the second portion of the duodenum and marked narrowing of the lumen of the third portion of the duodenum (A), and the largest part of the hematoma in diameter (B)**.

**Figure 3:**
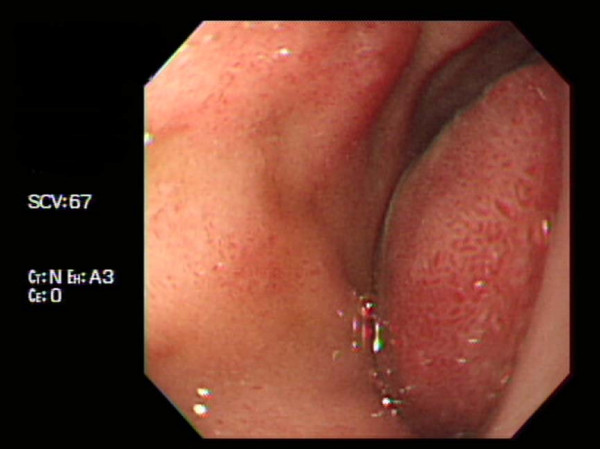
**Follow-up endoscopy shows a near-total obstruction of the duodenal lumen by the hematoma**.

**Figure 4:**
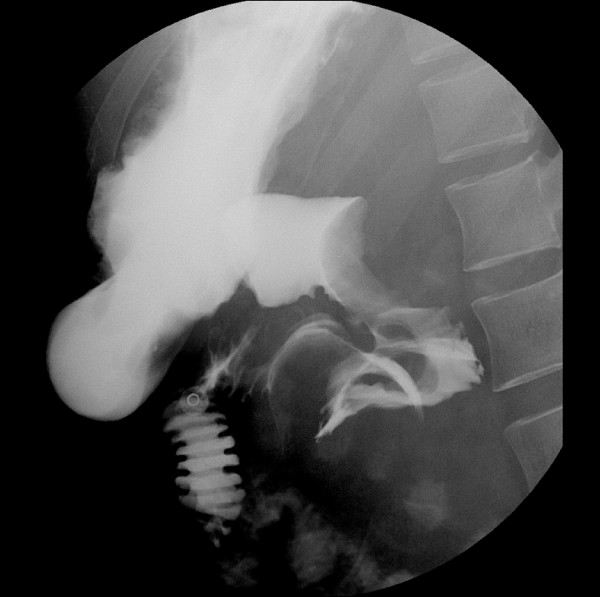
**Upper gastrointestinal series with gastrograffin reveals the hematoma compressing the second portion of the duodenum and the partial passage of gastrograffin via a near-totally occluded duodenal lumen without leakage outside**.

**Figure 5:**
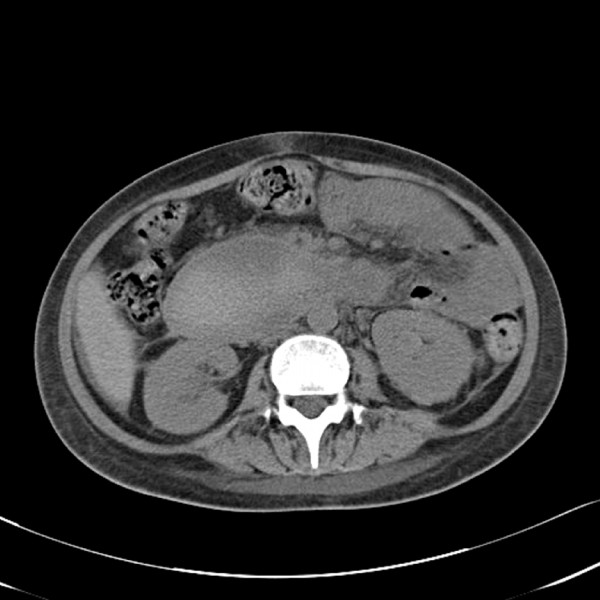
**Follow-up CT of the abdomen 4 weeks post-endoscopy demonstrates partial regression of the hematoma**.

**Figure 6:**
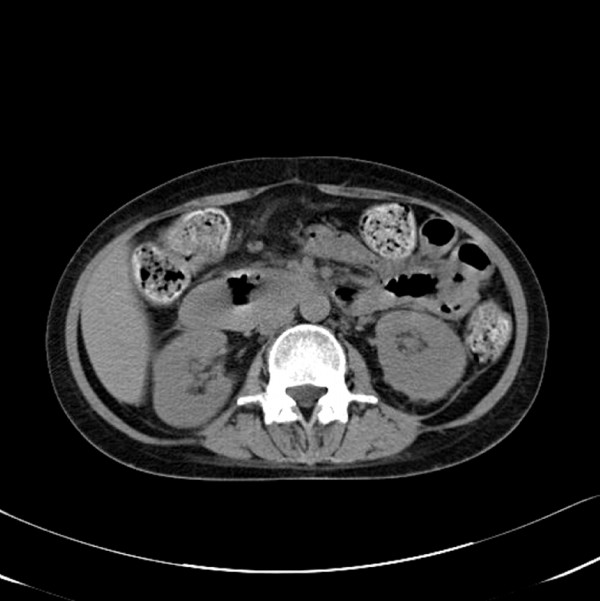
**Follow-up CT of the abdomen 12 weeks post-endoscopy demonstrates the decreased size of the hematoma**.

On the fourth day after the endoscopic treatment, the patient had diffuse abdominal distension and tenderness with persistent nausea and vomiting. An emergency computed tomography (CT) of the abdomen revealed a 13 × 6.5 cm mixed-density hematoma at the second and third portions of the duodenum with compression the ampulla of Vater, resulting in dilatation of the common bile duct and the intrahepatic ducts (Figure 2). In addition, there was a moderate amount of high-attenuation fluid in the perisplenic area, paracolic gutters and pelvic cavity, indicating a hemoperitoneum. Without inflammatory changes in the peripancreatic fat, to support a diagnosis of pancreatitis based on the CT findings, the serum amylase level had increased from 145 IU/L (normal, < 200 IU/L) on admission to 373 IU/L, with elevation of the serum lipase of 694 IU/L (normal, < 200 IU/L). The serum total and direct bilirubin values were 0.96 IU/L and 0.48 IU/L, respectively. A repeat endoscopy showed nearly complete obstruction of the lumen, at the site of treatment of the duodenal ulcer, by a very large hematoma (Figure 3). A diagnosis of duodenal obstruction, due to an intramural duodenal hematoma with hemoperitoneum, was made on the basis of the CT and follow-up endoscopic appearance and the progression of the anemia. The development of fever posed a therapeutic dilemma for the clinicians, who had to rapidly decide whether to perform a surgical evacuation or external drainage; the patient was conservatively managed with intravenous omeprazole and antibiotics, continuous nasogastric suction, and total parenteral nutrition. After 10 days, an upper gastrointestinal series using gastrograffin revealed a partial obstruction at the level of the second portion of the duodenum without definite evidence of leakage (Figure 4). Total parenteral nutrition was discontinued on the 14th day and the patient was slowly and carefully given liquids and then a soft meal. The patient was asymptomatic and was discharged shortly thereafter. Serial CT findings 4 weeks (Figure 5) and 12 weeks (Figure 6) after the onset of obstruction showed progressive resolution of the hematoma.

## Discussion

Intramural hematomas of the intestine usually present due to blunt trauma to the abdomen. In 60-80% of the reported cases the patients are children and young adults, and over 80% of the cases have a history of abdominal trauma [1,3,4]. An intramural hematoma of the abdomen is most commonly observed in the duodenal wall of the gastrointestinal tract. It is believed that the hematoma results from the bowel being crushed between the anterior abdominal wall and the vertebral column [3,4]. Because the duodenum is relatively fixed, it is therefore prone to this injury if enough force is applied to the anterior abdominal wall [4]. The rich submucosal vascular supply of the duodenum may also contribute to the development of a hematoma [3].

Nontraumatic intramural hematomas of the duodenum are reported to occur in some conditions such as pancreatic diseases, use of anticoagulants, and coagulation disorders [1,3-5]. Iatrogenic trauma caused by diagnostic or therapeutic endoscopy can also lead to an intramural duodenal hematoma. Although a local injection with epinephrine could cause tissue damage, in practice this does not appear to occur [1]. Underlying conditions, however, require special attention. Endoscopic biopsy or hemostasis may pose substantial risk to the subgroup of patients with leukemia, lymphoma, liver cirrhosis, and chronic renal failure [1,2,6,7].

As a manifestation secondary to the obstruction of the ampulla of Vater, due to the duodenal hematoma, pancreatitis can occur [1,4]. In addition, obstruction and subsequent dilatation of extra- or intrahepatic biliary tracts is probable [8]. In the present case, the serum amylase and lipase levels were elevated above the normal range. However, serum amylase and lipase concentrations have been reported to be moderately elevated in patients with chronic renal failure, and thus a serum amylase concentration of three times the upper limit of normal is often considered to be normal in such patients [9]. Therefore, interpretation of the results of the serum amylase and lipase in patients with chronic renal failure should proceed with caution.

Whereas in the past surgical evacuation or drainage was advised, current management of an intramural duodenal hematoma favors a conservative approach, including nasogastric suction and total parenteral nutrition [8,10-12]. Because the abundant blood supply of the duodenal wall is expected to absorb the hematoma promptly, the size of the hematoma does not appear to be a significant issue. Surgical management should be reserved for patients with a suspected perforation or with severely damaged intestines [10]. In the present patient, there may have been a small perforation due to the evidence of a hemoperitoneum. However, careful monitoring allowed for successful conservative management.

Two prior cases with nontraumatic duodenal hematomas have been reported as complications of endoscopic treatment in patients with ESRD [1,2]. Those case reports did not describe in detail the causes of the bleeding diatheses. In fact, the pathogenesis of the hemorrhagic diathesis, in patients with ESRD, is likely multifactorial and cannot be explained on the basis of a single mechanism. Platelet dysfunction, anemia, dialysis, the accumulation of medications due to poor clearance, anticoagulation used during hemodialysis, and the hemodialysis process itself play some role in causing impaired hemostasis in patients with ESRD [13]. In the present case there was associated coagulopathy-causing factors such as platelet dysfunction, thrombocytopenia, anemia, use of anticoagulants, and hemodialysis. In such cases, before an invasive procedure, the administration of desmopressin acetate or conjugated estrogen, achieving a hematocrit of 30%, might reduce the risk of bleeding in patients with ESRD [13].

## Conclusion

This report describes an uncommon case of intramural hematoma of the duodenum following endoscopic hemostasis in a patient with chronic renal failure on maintenance hemodialysis. The onset of symptoms such as abdominal pain and vomiting and progression of anemia should suggest the possibility of complications after endoscopy. Once the diagnosis is confirmed clinically and/or by laboratory and imaging findings, conservative treatment with nasogastric suction and parenteral nutrition can be successfully used as treatment for hemodynamically stable patients. Careful monitoring for the emergence of complications after endoscopic procedures is needed especially in patients with a coagulopathy.

## Abbreviations

CT: computed tomography;

## Consent

Written informed consent was obtained from the patient for publication of this case report and accompanying images. A copy of the written consent is available for review by the Editor-in-Chief of this journal.

## Competing interests

The authors declare that they have no competing interests.

## Authors' contributions

SC, CWP and YSC were involved in the day to day patient care. HWC and SJS performed the literature review. SC and YSC were major contributors in writing the manuscript. All authors read and approved the final manuscript.
